# Market return spillover from the US to the Asia-Pacific Countries: The Role of Geopolitical Risk and the Information & Communication Technologies

**DOI:** 10.1371/journal.pone.0290680

**Published:** 2023-12-14

**Authors:** Minh Phuoc-Bao Tran, Duc Hong Vo

**Affiliations:** Research Centre in Business, Economics, and Resources, Ho Chi Minh City Open University Vietnam, Ho Chi Minh City, Vietnam; Universiti Teknologi MARA, MALAYSIA

## Abstract

This study examines the market return spillovers from the US market to 10 Asia-Pacific stock markets, accounting for approximately 91 per cent of the region’s GDP from 1991 to 2022. Our findings indicate an increased return spillover from the US stock market to the Asia-Pacific stock market over time, particularly after major global events such as the 1997 Asian and the 2008 global financial crises, the 2015 China stock market crash, and the COVID-19 pandemic. The 2008 global financial crisis had the most substantial impact on these events. In addition, the findings also indicate that US economic policy uncertainty and US geopolitical risk significantly affect spillovers from the US to the Asia-Pacific markets. In contrast, the geopolitical risk of Asia-Pacific countries reduces these spillovers. The study also highlights the significant impact of information and communication technologies (ICT) on these spillovers. Given the increasing integration of global financial markets, the findings of this research are expected to provide valuable policy implications for investors and policymakers.

## Introduction

Since the 2008 global recession, scholars have had significant debates about the effect of the US stock market on Asia-Pacific stock markets. There are two contrasting views on this effect. One view asserts that the Asia-Pacific markets are less affected by US economic shocks because their economic growth is not reliant on the US [1,2]. Meanwhile, another group of scholars argues that the Asia-Pacific markets depend on the US stock market owing to their relatively emerging size [3,4]. Based on this divergence, our study aims to investigate the spillovers from the US stock market to Asia–Pacific stock markets.

The literature offers two theories to explain the spillovers between stock markets. These theories are not mutually exclusive and provide complementary explanations. The first group emphasizes the interconnectedness of economies through real and financial linkages. When a crisis occurs in one country, it can transmit to other countries due to the interdependence of their economic fundamentals. Cross-border flows of goods, services, and capital significantly enlarge spillovers [5,6]. The second group of theories highlights market imperfections and the behaviour of international investors as drivers of spillovers [7–9]. Information asymmetries cause uncertainty among investors regarding the economic fundamentals of a country. As such, when a crisis occurs in one country, it signals international investors to reassess risks in other countries. Inexperienced or less informed investors may struggle to distinguish the informed signals from falling prices and follow the strategies of better-informed investors, leading to excess co-movements across markets [10,11].

In addition, technology has played an essential role in modern economies. Countries globally have now relied on information technology as an effective tool to secure sustainable economic growth and social transformation. Information technology has also affected geopolitical risk to a certain extent. Superpower countries like the US, China, Russia, and India now rely on information technology to support their political, military and economic advantages. However, the influence of US geopolitical risk and the information and communication technologies (ICT) on the spillovers among stock markets and spillover from the US stock market to Asia-Pacific stock markets, in particular, has largely been neglected in previous studies. Geopolitical risk is related to wars, terrorism and tensions among countries that adversely affect the normal course of international relations and peace [12]. Geopolitical risk has recently attracted attention from scholars since the emergence of the Russia—Ukraine conflict in February 2022 and is considered a crucial factor affecting investment decisions, which ultimately impacts international capital flow [13]. Meanwhile, information and communication technologies (ICT) refers to various technological instruments and materials utilized for disseminating, storing, generating, distributing, or exchanging information. ICT is another important factor in global stock markets’ spillovers due to enabling unrestricted sharing of information and data, reducing market inefficiencies such as information asymmetries [14,15]. As such, geopolitical risk and ICT are expected to significantly impact spillover between stock markets.

Based on these considerations, we use the TVP-VAR-based connectedness approach to investigate the spillovers from the US to the Asia-Pacific stock markets. Our analysis covers key events that the world stock markets have experienced, such as the 1997 Asian financial crisis, the 2008 global financial crisis, and the 2020 COVID-19 pandemic. We also conduct further analysis to evaluate the effect of US geopolitical risk, US economic policy uncertainty and various Asia-Pacific countries’ domestic factors, including local geopolitical risk, internet coverage, financial openness, and trade openness on the spillovers from the US stock market to Asia-Pacific stock markets.

The contributions of this paper to the existing literature are twofold. First, to the best of our knowledge, this is one of the first studies to examine the geopolitical risk and the ICT on spillovers across stock markets, particularly the Asia-Pacific region. Second, we provide fresh evidence on the dynamic spillover effect from the US to Asia-Pacific stock markets using the TVP-VAR-based connectedness approach. This approach offers many advantages, such as overcoming arbitrary choice of rolling-window size and losing valuable observations compared to its predecessor, the VAR-based connectedness approach.

Following this introduction, the remainder of this paper is structured as follows. The next section briefly reviews relevant literature on stock market spillovers. The research method and data are presented in the following section. We then present and discuss empirical results from this analysis, followed by the concluding remarks and implications.

## Literature review

A large body of research agrees that the stock market reflects macroeconomic fundamentals because it reflects the market’s expectations about future business prospects. For instance, Bomfim [16] states that the volatility of US stocks rises notably following unexpected monetary policy announcements. On the other hand, UK stock market volatility tends to decrease during macroeconomic announcements [17]. Also, Saba Qureshi et al. [18] present a transmission of risk from exchange rates to sectoral returns in the Pakistan stock market from 1992 to 2017. This is because the fluctuations observed in the domestic currency directly affect the competitive position of local companies on the global stage, subsequently affecting their cash flows and the stock market [19]. Arshanapalli et al. [20] consider if macroeconomic news affects the varying volatility and covariance, indicating changing risk premiums in the US stock and bond markets. They find that stock and bond market volatility rises on the day of these announcements. Likewise, Fuss et al. [21] investigate the connection between macroeconomic events in Germany and the US and two implied volatility indices, namely the DAX Volatility Index (VDAX) and the Chicago Board Options Exchange Volatility Index (VIX). Their findings reveal that on days when macroeconomic announcements are released, the VDAX and VIX indices decline. These responses are particularly significant during the 2008 global financial crisis period. As such, the downward (upward) of the US market reflects the outlook of the US macroeconomy to go down (up). On the other hand, the US is one of the largest import partners of the Asia-Pacific countries [22]. As such, the outlook of the US economy significantly affects the outlook of the Asia-Pacific economies. This view is consistent with King and Wadhwani [9] and Goldstein [23], as investors tend to re-evaluate the stock market outlooks through changes in another stock market, especially after the outbreak of financial crises. Such re-assessment, in turn, leads to fluctuations in Asia-Pacific stock markets. On this basis, we argue that:

H_1_ *The US stock market significantly influences the Asia-Pacific stock market*, *especially after major shocks*.

The geopolitical risk is documented to heavily affect the financial markets in general and stock markets in particular. It is often considered a major factor influencing investment decisions by central bankers, business investors, and the financial press [12,24,25]. Such an increase in geopolitical risk and uncertainty can affect stock markets by triggering risk aversion among investors. Brennan and Cao [26] developed a model for international equity portfolio investment flows based on differences in informational endowments between foreign and domestic investors. They argue that due to rising global uncertainty, there is a difference in the quantity and quality of information available to domestic and foreign individuals, causing them to favour domestic assets. In other words, the information asymmetry between domestic and international investors due to a significant increase in uncertainty (i.e., geopolitical risk) in the recipient country of the capital inflow (i.e., Asia-Pacific stock markets) can significantly reduce cross-border investment activities of US investors, which therefore reduces spillovers from US stock market to Asia-Pacific stock markets. On the contrary, a significant increase in US uncertainties, such as economic policy and geopolitical tensions, increases spillovers from the US stock market to Asia-Pacific stock markets since increased uncertainties can adversely influence the performance of investments in the US, causing investors to shift their portfolios away from the US. Based on this reasoning, we argue that:

H_2a_ *As the uncertainty (i*.*e*., *geopolitical risk) in Asia-Pacific countries increases*, *the spillovers from the US stock market to the Asia-Pacific stock markets decrease*.
H_2b_ *As the US uncertainties (i*.*e*., *economic policy uncertainty and geopolitical risk) in Asia-Pacific countries increase the spillovers from the US stock market to the Asia-Pacific stock markets increase*.

Trade plays a critical role in connecting the two markets [27]. Increased trade can increase a country’s exposure to external impacts by reducing exports, which make up a significant portion of a country’s overall output, and, therefore, significantly affect the stock markets. Financial openness is another plausible factor in exacerbating contagious effects between two stock markets. It is because when a country with foreign assets faces a quick deterioration in a balance sheet in the event of unexpected adverse shocks from outside the country [28]. On this basis, we hypothesize:

H_3_ *The larger the trade and financial openness of Asia-Pacific countries*, *the more significant the spillovers from the US stock market to a country’s stock market*.

ICT is another factor in enabling the US market shock to spread to Asia-Pacific stock markets since financial markets are actually “information markets” [29,30]. Accordingly, ICT enables the widespread availability and high speed of information in financial markets. It, therefore, reduces issues such as time delays and information asymmetries which can result in market failures. However, the explosive growth of the ICT also contributes a large part to the increasing volatility of financial markets [31] since the ICT allows faster and more efficient execution of trades across different markets, reducing the time it takes for information to flow from one market to another. As a result, investors can respond more quickly to changes in the market and revise their investment strategies accordingly. In other words, any significant movement in the US stock market will likely affect the Asian market almost instantly, which motivates us to argue that:

H_4_ *The higher the level of ICT development a country has*, *the more significant the spillovers from the US stock market to the country’s stock market*.

Various empirical studies have examined the effects of geopolitical risk on individual markets. However, no study examines the effect of geopolitical risk on the spillover from one market to another, although the global financial market is increasingly integrated. For example, the inverse relationship between country-specific geopolitical risk and stock market returns has been confirmed by Yang and Yang [24] and Hoque and Zaidi [32]. Furthermore, Bouras et al. [33] also note that the global GPR provides a stronger and more positive effect on emerging market volatility than the effect of the country-specific GPR from 1998 to 2017. However, Choi [34] uses the wavelet coherence analysis to examine the effects of the global and Korean GPR on the stock market volatility of three North-East Asian countries, namely Korean, Japanese, and Chinese stock markets, from July 1997 to May 2021. Their empirical results show that Korean GPR is positively correlated with South Korean and Japanese stock market volatility, while the influence of global GPR on these three markets is unclear. Likewise, previous studies mainly focus on the impact of ICT on stock market participation [35,36], stock market development [37], or stock market performance [38] while ignoring its impact on spillovers between stock markets.

The connectedness between the US and Asia-Pacific stock markets has been underexamined in the existing literature. Employing GARCH-BEKK, Li and Giles [39] show significant spillovers from the US stock market to Japan and six Asian developing countries, including China, India, Indonesia, Malaysia, the Philippines and Thailand, from January 1993 to December 2012. The results indicate one-way shock and volatility transmissions from the US market to the Japanese and Asian emerging markets. Additionally, during the Asian financial crisis, bidirectional volatility spillovers between the US and Asian markets are observed. Emawtee et al. [40] adopted the FIVAR-based connectedness approach to assessing the stock market volatility spillovers between three countries, the United States, China, and Australia, from July 2007 to May 2016. The study shows a unidirectional volatility spillover from the United States to China in the financial services, industrials, consumer discretionary, and utilities sectors. On the other hand, the volatility spillover from the Australian stock market to the Chinese stock market is not significant in the financial services, telecommunications, and energy sectors. However, a bilateral relationship across all industries in the three countries is confirmed when the global financial crisis is excluded from the sample. Also, Park [41] adopts the VAR-based connectedness approach to examine the spillovers among Asia-Pacific stock markets from March 2002 to March 2019. The empirical findings indicate that after the 2008 global financial crisis, the US is considered an overwhelming volatility transmitter to the Asia-Pacific markets, while Korea is the net receiver of shocks transmitted from other markets.

Unlike previous studies, our sample is enlarged by examining spillovers from the US stock market to 10 Asia-Pacific countries, including Australia, China, Hong Kong, India, Japan, Korea, Malaysia, the Philippines, and Thailand. Our sample includes both developed and developing stock markets. These countries account for approximately 91 per cent of the Asia-Pacific GDP. Examining the spillover effects between the world’s most developed stock market, the US, and other developed markets (including Australia, Hong Kong, and Japan) and emerging markets (China, India, Korea, Malaysia, the Philippines, and Thailand) is crucial for investors. If the integration between emerging and developed financial markets is weak, investors in developed markets can diversify their portfolios by including stocks from the emerging market, thereby reducing risk. However, if emerging stock markets are fully integrated with developed markets, the benefits of a risk diversification strategy from holding emerging market assets disappear. On the other hand, unlike previous studies, our study uses the TVP-VAR-based connectedness approach to examine spillovers from the US stock market to the Asia-Pacific stock markets. Our study is related to, but different from, Park [41] because our study adopts an improved version of VAR-based connectedness, the TVP-VAR-based connectedness approach. The TVP-VAR-based connectedness approach is considered superior to the VAR-based connectedness approach because it is not influenced by the subjective determination of the window size, which may lead to unreliable estimates. Moreover, our study is one of the first to examine the effect of geopolitical risk and information and communication technology (ICT) on spillovers among stock markets. Investigating the impact of geopolitical risk on spillovers is crucial, especially considering the heightened geopolitical tensions caused by events like the Russo-Ukrainian conflict [42] and the US-China tension [43]. Additionally, the role of ICT should not be overlooked as it can significantly reduce market inefficiencies such as information disparities and delays, ultimately affecting stock market performance [14]. Examining the effects of ICT on the transmission of spillovers between stock markets can provide valuable insights for policymakers, aiding in the development of stock markets and improving a nation’s competitiveness in attracting capital flows. This, in turn, contributes significantly to poverty reduction and economic development.

## Data and research methodology

### Data

We employ monthly data from 10 Asia-Pacific stock market indices to estimate the market returns. These 10 Asia-Pacific stock markets are Australia, China, Hong Kong, India, Japan, Korea, Malaysia, the Philippines, and Thailand, accounting for approximately 91% of Asia-Pacific GDP (nominal) according to the IMF’s October 2022 World Economic Outlook. The market returns of each country at month t, *RET*_*t*_, are calculated as $RET_{t}=\ln\left(\frac{P_{t}}{P_{t-1}}\right),$where *P*_*t*_ is the closed price of month *t*. Furthermore, to examine the effect of geopolitical risk and economic policy uncertainty on the stock return spillovers from the US to Asia-Pacific stock markets, a set of variables are also employed, including the US geopolitical risk index (GPR) developed by Caldara and Iacoviello [12]; the US’s economic policy uncertainty index (EPU) developed by Baker et al. [44] along with trade openness; financial openness; proportion of individuals who used internet from any location in the last three months as a proxy of the ICT; and real GDP growth. Our data spans from January 1991 to November 2022. The details of the variables and their data sources can be found in the appendix (Table A1 in S1 File). Table 1 provides descriptive statistics.

**Table 1:** Descriptive statistics of stock market returns.

Series	Mean	Maximum	Minimum	Std. Dev.	Skewness	Kurtosis	JB test	ADF	PP
Australia	0.005	0.095	-0.242	0.040	-1.166	6.851	322.6***	-19.280***	-19.283***
China	0.008	1.020	-0.373	0.117	2.548	24.557	7810***	-20.021***	-20.016***
Hong Kong	0.005	0.265	-0.348	0.070	-0.199	5.613	111.2***	-18.764***	-18.751***
India	0.011	0.351	-0.273	0.076	-0.016	5.382	90.3***	-18.166***	-18.161***
Indonesia	0.008	0.250	-0.379	0.073	-1.076	8.235	509.9***	-16.137***	-16.036***
Japan	0.000	0.150	-0.272	0.058	-0.503	3.936	30.08***	-18.472***	-18.504***
S.Korea	0.004	0.411	-0.318	0.074	0.214	6.595	208.6***	-17.643***	-17.597***
Malaysia	0.003	0.294	-0.285	0.061	0.039	7.966	392.6***	-17.596***	-17.593***
Philippines	0.006	0.332	-0.299	0.072	-0.082	6.466	191.7***	-18.414***	-18.407***
Thailand	0.002	0.284	-0.359	0.079	-0.188	5.860	132.4***	-18.466***	-18.473***
US	0.006	0.119	-0.186	0.043	-0.754	4.522	73.07***	-19.524***	-19.531***

Note: The first column presents the stock market return of each country. “Std. Dev.” stands for standard deviation. The Jarque-Bera test (JB test) is adopted to test the null hypothesis of normality; Augmented Dickey-Fuller (ADF) and Phillips–Perron (PP) tests are employed for testing the null hypothesis of a unit root in the series.

*** indicates the level of significance at 1 per cent.

**Fig 1:**
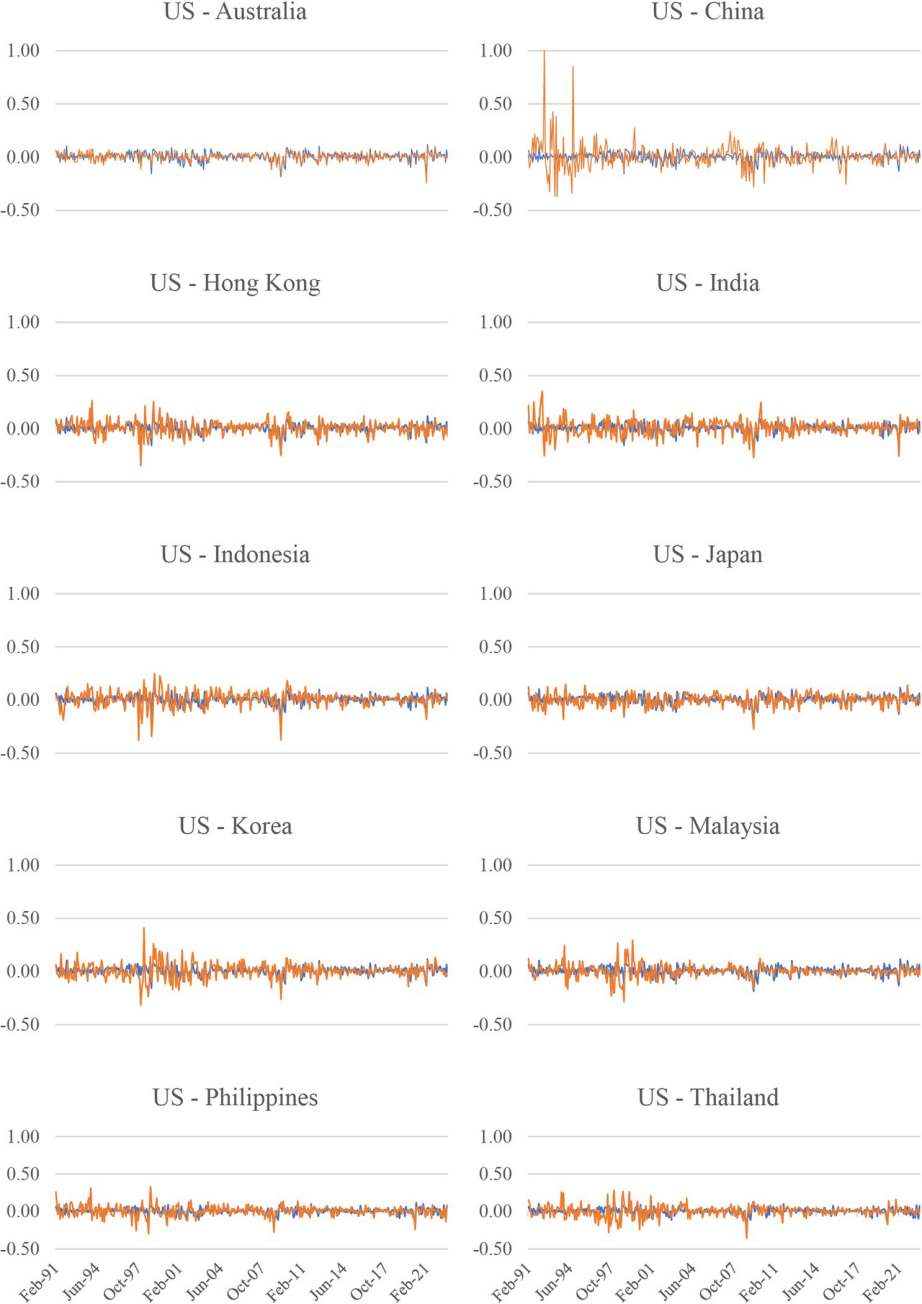
Stock market returns across stock markets in the Asia-Pacific region and the US. Note: While the vertical axis is the value of stock market returns (%), the horizontal axis is the month. The blue line illustrates the US stock market return, and the orange line represents each Asia-Pacific stock market’s return.

The US and the Asia-Pacific stock market returns are depicted in Fig 1. The blue line represents the return of the US stock market, while the orange line represents the return of each Asia-Pacific stock market. First, during the 1997 Asian financial crisis, most Asia-Pacific stock markets, including Hong Kong, Indonesia, Korea, the Philippines, Malaysia, and Thailand, experienced significant volatility, while the US market was relatively less volatile. Second, major upheavals, such as the 1997 Asian financial crisis and the 2008 global financial crisis, led to significant drops in stock market returns across countries in the Asia-Pacific region. On the other hand, the impacts of the 2015 China stock market crash and the COVID-19 pandemic are relatively less pronounced, except for the significant impact of the 2015 China stock market crash on the Chinese stock market return.

### Research methodology

#### TVP-VAR-based connectedness approach

Our analysis adopts the TVP-VAR-based connectedness approach developed by Antonakakis et al. [45] to measure how much stock returns have spilled over from the US to the Asia-Pacific markets. This method is grounded in the work of Diebold and Yilmaz in 2009 [46], which was extended by Diebold and Yılmaz in 2014 [47]. Various modifications to the connectedness approach have emerged since its inception. The TVP-VAR-based approach, introduced by Antonakakis et al. [45], combines Diebold and Yılmaz [47]’s approach with the time-varying parameter vector autoregressive (TVP-VAR) model developed by Koop and Korobilis [48]. This approach has advantages over other connectedness approaches, as it is not affected by subjective window size determination and can retain valuable data observations when estimating the variance-covariance matrix using the Kalman filter procedure. The TVP-VAR model based on the Bayesian information criterion (BIC) has a specific form.


$$\Delta y_{t}=B_{t}\Delta y_{t-1}+u_{t}\;with\;u_{t}\sim N(0,\vartheta_{t})$$ (1)

$$vec\left(B_{t}\right)=vec\left(B_{t-1}\right)+v_{t}\;with\;v_{t}∼N\left(0,{\text{R}}_{t}\right)$$ (2)

Where Δ*y*_*t*_ and Δ*y*_*t*-1_ are *n* × 1 vectors while *B*_*t*_ and ϑ_*t*_ are *n* × *n* dimensional matrices. *vec*(*B*_*t*_) and *vec*(*B*_*t*-1_) are *n*^2^ × 1 vectors. *R*_*t*_ is the *n*^2^ × *n*^2^ matrix. ϑ_*t*_ and *R*_*t*_ are time-varying variance-covariance matrices. n = 11 is the number of stock markets in our sample.

Following the work of Koop et al. [49] and Pesaran and Shin [50], Diebold and Yılmaz [47] improve their connectedness approach by computing the scaled generalized forecast error variance decomposition (GFEVD) using an H-step-ahead forecast horizon. The calculations are made in a way unaffected by the variables’ order. Consistent with Diebold and Yılmaz [47], Antonakakis et al. [45] utilize the Wold theorem to transform a time-varying parameter vector autoregression (TVP-VAR) into its corresponding vector moving average representation, also known as TVP-VMA. The transformation allows them to obtain the generalized impulse response function (GIRF) and generalized forecasting error variance decomposition (GFEVD):

$$\Delta y_{t}=\sum_{i=1}^{p}B_{it}\Delta y_{t-i}+u_{t}=\sum_{j=0}^{\infty }A_{jt}u_{t-j}$$ (3)

Where *A*_*jt*_ is *n* × *n* dimensional matrix, then GIRFs, ${ℶ}_{ij,t}^{g}$(H), which is the response of stock market j as a result of a shock in stock market i, is computed by the differences between an H-step-ahead forecast of two cases that are once stock market i is shocked, and once stock market i is not shocked.

Next, the GFEVD (*H*) is calculated to represent the return volatility spillover from the US stock market (j) to each Asia-Pacific stock market i. It shows the extent to which variable j influences variable i in terms of its forecast error variance share. The GFEVD, $\tilde{\omega }$_*ij*,*t*_(*H*) has the following form:

$${\tilde{\omega }}_{ij,t}(H)=\frac{\sum_{t=1}^{H-1}{ℶ}_{ij,t}^{2}}{\sum_{j=1}^{n}\sum_{t=1}^{H-1}{ℶ}_{ij,t}^{2}}$$ (4)

Where $\sum_{j=1}^{n}{\tilde{\omega }}_{ij,t}(H)=1$ and $\sum_{i,j=1}^{n}{\tilde{\omega }}_{ij,t}(H)=n$. Based on the GFEVD, the spillover from the US stock market (j) to all the Asia-Pacific stock markets i, Spillover_i←j,t_, is defined as:

$$\;TO\;OTHERS\;Spillove{r\;}_{i\leftarrow j,t}(\text{H})=\frac{\sum_{i=1,i\neq j}^{n}{\tilde{\omega }}_{ij,t}(H)}{\sum_{j,i=1}^{n}{\tilde{\omega }}_{ij,t}(H)}\times 100$$ (5)

Meanwhile, *the net pairwise spillover between the US stock market (j) and each of the Asia-Pacific stock market i*, *Spillover*_*ij*,*t*_ is defined as:

$$\;NET\;PAIRWISE\;Spillove{r\;}_{ij,t}=\;Spillove{r\;}_{i\to j,t}-\;Spillove{r\;}_{i\leftarrow j,t}$$ (6)

#### The impact of US geopolitical risk and US economic policy uncertainty on spillovers from the US stock market to Asia-Pacific stock markets

To examine the determinants of the spillovers from the US to Asia-Pacific stock markets, our model is as follows:

$$\;Spillove{r\;}_{ij,t}=\alpha_{i}+\beta_{t}+GPR_{t}+US\;GPR_{t}+US\;EPU_{t}+\;InternetCoverag{e\;}_{i,t}+FO_{i,t}+TO_{i,t}+\;Real\;GDP\;growt{h\;}_{i,t}$$ (7)

Where *Spillover*_*ij*,*t*_ is the spillovers from the US stock market (j) to the Asia-Pacific stock markets (i) or “TO OTHERS” estimated from Eq (5). *α*_*i*_ is country-fixed effect to capture market-specific factors that affect both geopolitical risk and spillovers from US stock market such as political influence. At the same time, *β*_*t*_ is a year-fixed effect to control structural breaks caused by crises, as indicated by the literature. *GPR*_*t*_ is the geopolitical risk of Asia-Pacific countries; *US GPR*_*t*_ is the US geopolitical risk. *US EPU*_*t*_ is the US economic policy uncertainty. *Spillover*_*ij*,*t*_,*GPR*_*t*_,*US GPR*_*t*_, and *US GPR*_*t*_ are all averaged to convert from monthly to annual frequency (year t). Following Marszk and Lechman [14], we employ a proportion of individuals who used the internet from any location in the last three months in year t, *ICT*_*i*,*t*_, as a proxy of the ICT. *FO*_*i*,*t*_ and *TO*_*i*,*t*_ are financial openness (% of GDP) and trade openness (% of GDP). *Real GDP growth*_*i*,*t*_ is the % real GDP growth of country i in year t. Descriptive statistics of the model (7) can be found in Table 2 below.

**Table 2:** Descriptive statistics of the model (7).

Variable	Mean	Max	Min	Std. Dev.
Spillover	30.115	72.247	0.020	17.909
Local GPR	14.153	91.671	1.421	15.703
US GPR	225.303	434.971	105.397	80.650
US EPU	112.504	242.987	71.329	36.511
ICT	33.080	96.505	0.000	32.847
Financial Openness	0.434	2.311	-1.927	1.394
Trade Openness	94.156	442.620	15.810	90.473
Real GDP growth	4.523	-13.100	14.300	3.898

Note: “Spillover” stands for the spillovers from the US stock market to each of the Asia-Pacific stock markets; “GPR” stands for geopolitical risk; “EPU” represents economic policy uncertainty, “ICT” is the proportion of individuals who use the internet, and "GDP" is a gross domestic product. "Std. Dev." stands for standard deviation.

## Empirical results

### Static spillovers across stock markets

The average results of the interlinkages between stock markets are reported in Table 3. In this table, the first row (in bold) indicates where the spillover is transmitted, while the first column (in bold) indicates where the spillover is received. For example, the pair Australia—S&P 500 has a value of 13.39, which means that 13.39% of the fluctuations in a unit of the Australian stock market return is from the return on the US stock market (S&P 500). The last row (To) represents the degree to which the stock market in that column transmits to the remaining stock markets. The last column (From) represents the degree to which the stock market in that column receives spillover from the remaining stock markets.

**Table 3:** Total return spillovers between the US stock market and ten markets in the Asia-Pacific region, January 1985 to November 2022.

	The US	Australia	China	Hong Kong	India	Indonesia	Japan	Korea	Malaysia	Philippines	Thailand	FROM
SP500	35.84	12.47	0.72	11.99	2.98	5.23	7.15	6.01	5.49	7.19	4.94	64.16
Australia	**13.39**	38.76	0.81	9.85	3.06	3.97	7.48	5.58	5.56	7.86	3.68	61.24
China	1.75	1.89	82.61	4.12	1.86	1.07	1.65	1.41	1.39	1.44	0.81	17.39
Hong Kong	**10.43**	8.05	1.62	30.44	3.39	7.43	4.06	6.75	10.34	9.68	7.83	69.56
India	4.29	4.12	1.08	5.33	62.61	4.7	2.93	3.66	2.68	2.76	5.83	37.39
Indonesia	5.49	3.99	0.48	8.95	3.43	36.8	3.7	5.31	8.04	11.05	12.76	63.2
Japan	**9.65**	9.48	0.87	5.97	2.67	4.65	50.24	8.71	1.41	2.98	3.37	49.76
Korea	6.88	5.95	0.69	8.78	2.87	5.75	7.38	42.35	4.43	4.52	10.38	57.65
Malaysia	5.82	5.39	0.71	12.44	2.22	8.28	1.33	4.27	36.93	12.84	9.78	63.07
Philippines	6.89	6.93	0.65	10.65	2.17	10.35	2.3	4.01	11.76	33.69	10.58	66.31
Thailand	4.99	3.55	0.35	8.91	3.82	12.11	2.65	8.92	9.13	10.71	34.85	65.15
**TO**	**69.59**	61.82	7.98	86.98	28.47	63.54	40.63	54.63	60.23	71.04	69.96	TCI: 55.90%

As presented in Table 3, the spillover from S&P 500 to Asia-Pacific stock markets is quite high (69.59 per cent). The S&P 500 index explains movements in developed markets in the Asia-Pacific region the most: Australia (13.39 per cent), Hong Kong (10.43%), and Japan (9.65 per cent). Finally, the total connectedness index (TCI) is 55.9 per cent, implying that the interactions among 11 stock markets in our sample explain 55.9 per cent of the variance in the network, leaving approximately 44 per cent of error variance due to idiosyncratic effects.

### Dynamic spillovers across stock markets

The spillover analysis mentioned earlier provides an initial insight into the interaction among stock markets over the full sample period. However, it is important to note that the analysis above presents a static view and does not illustrate how the spillovers of the market risk have changed over time, as such dynamic analyses are adopted in this section.

**Fig 2:**
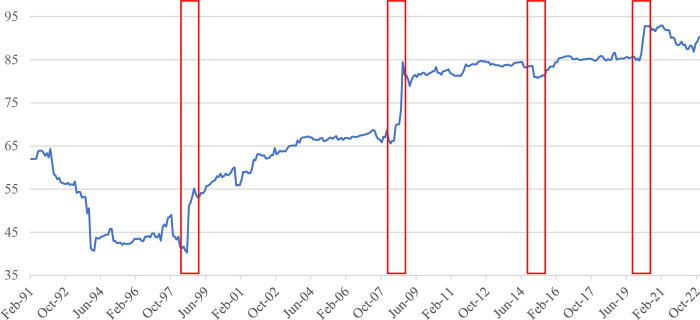
Spillover from the US stock market to all Asia–Pacific stock markets. Notes: Estimated based on TVP-VAR approach with a lag length of 1 (BIC) and one step-ahead forecast horizon.

Fig 2 illustrates how the US stock market returns spill over into all Asia-Pacific stock markets included in our sample. The spillover tends to increase around major crises such as the 1997 Asian financial crisis, the 2008 global financial crisis, 2015 China’s stock market crash and most recently, the health crisis induced by the COVID-19 pandemic in 2020. Overall, the spillovers from the US stock market to Asia-Pacific stock markets during the COVID-19 pandemic are relatively weak compared to other major events. The results of our study align with the research conducted by Park [41], who utilizes a VAR-based connectedness approach to investigate the dynamics among eighteen member economies of the Asia-Pacific Economic Cooperation (APEC) from March 2002 to March 2019. Park’s study finds that the 2008 global financial crisis significantly increased the spillovers from the United States to Asian markets. This outcome reinforces the notion that the interdependence between the US and Asian economies has profound implications for regional financial stability. Moreover, the findings are also consistent with the findings from Youssef et al. [51]), who also employ a TVP-VAR-based connectedness approach to examine the spillovers among eight stock markets in China, Italy, France, Germany, Spain, Russia, the US, and the UK. Their study focuses on the period from the 1^st^ of January 2015 to the 18^th^ of May 2020. Youssef et al. [51] indicate that the spillovers among these countries tend to surge around the 2015 China stock market crash and the 2020 COVID-19 pandemic. Overall, the findings support our Hypothesis 1. We also note that the findings are also consistent with the incomplete information model from King and Wadhwani [9] and the wake-up call hypothesis from Goldstein [23], implying that fluctuations in the US stock market cause investors to re-evaluate the Asia-Pacific stock market fundamentals and thus result in fluctuations in the Asia-Pacific stock markets.

Findings from various studies have indicated that the spillover effect between mature financial markets is significant [52–54], whereas the spillover effect among emerging markets tends to be weaker [54,55]. However, the spillovers between developed and emerging markets have been largely underexamined. As such, this motivates us to examine the spillover from the US, the largest financial market globally, to developed and emerging markets in Asia. This is significant because, according to risk diversification theory [56], holding stocks in markets with a strong relationship or spillover effect on each other is not beneficial in the long run. As such, examining how developed and emerging markets in Asia respond to volatility in the US market is important in providing valuable implications for firms and practitioners to minimize their risk.

**Fig 3:**
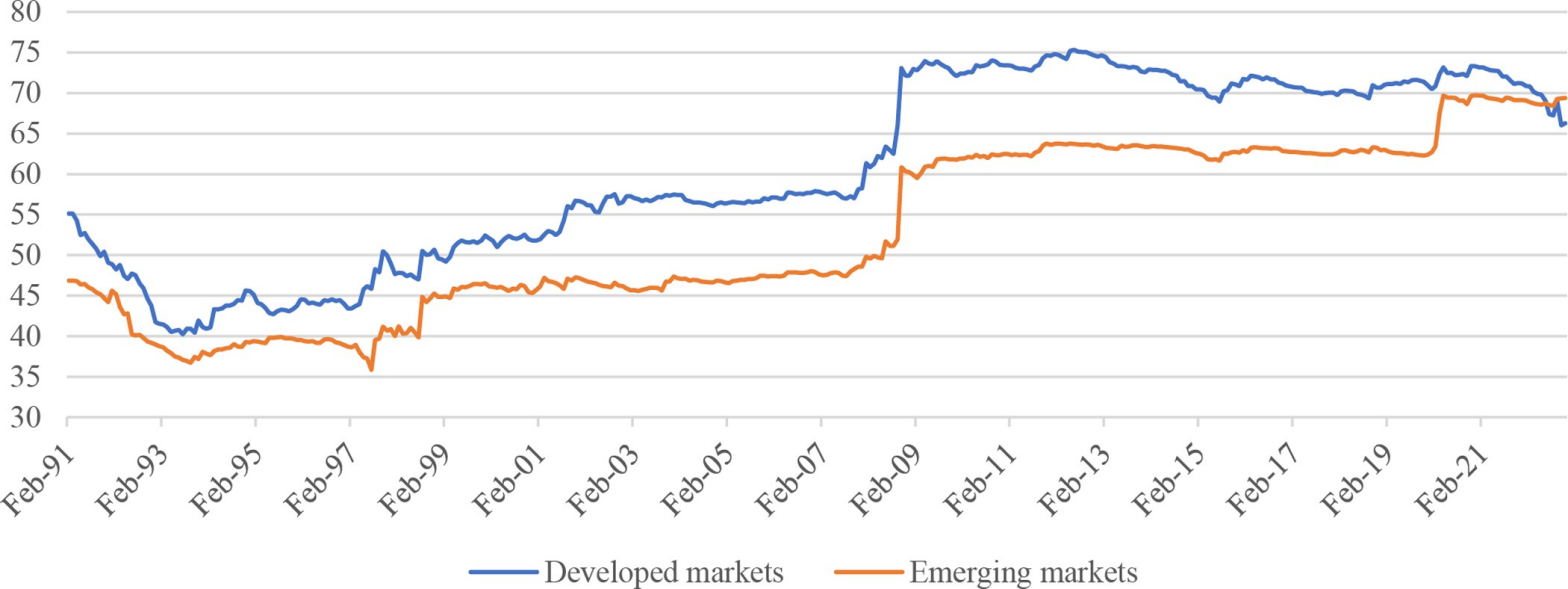
Spillovers from US’s S&P 500 to Asia–Pacific’s emerging and developed stock markets. Notes: Estimated based on TVP-VAR approach with a lag length of 1 (BIC) and one step-ahead forecast horizon. Developed Asian markets include Australia, Hong Kong, and Japan, while emerging Asian markets includes China, India, Korea, Malaysia, the Philippines, and Thailand.

Based on the 2023 MSCI Annual Market Classification [57], our sample consists of three developed markets in the Asia-Pacific region: Australia, Hong Kong, and Japan, whereas seven emerging markets include China, India, Korea, Malaysia, the Philippines, and Thailand. Fig 3 displays the results of the spillover of return volatility from the US market to the developed group of Asia-Pacific markets (represented by the blue line) and the emerging group of Asia-Pacific markets (represented by the orange line). Overall, during the entire period, the spillover of return volatility from the US to the developed Asian markets group is higher than the emerging markets. Following the 2008 financial crisis, the spillover from the US to the developed markets in the Asia-Pacific region varies between 70 and 75 per cent, while the spillover to the emerging markets remained around 60 to 65 per cent from 2009 to the end of 2019. However, after the outbreak of the COVID-19 pandemic, the spillover from the US to the emerging markets increased substantially, reaching the level observed in the developed markets.

**Fig 4:**
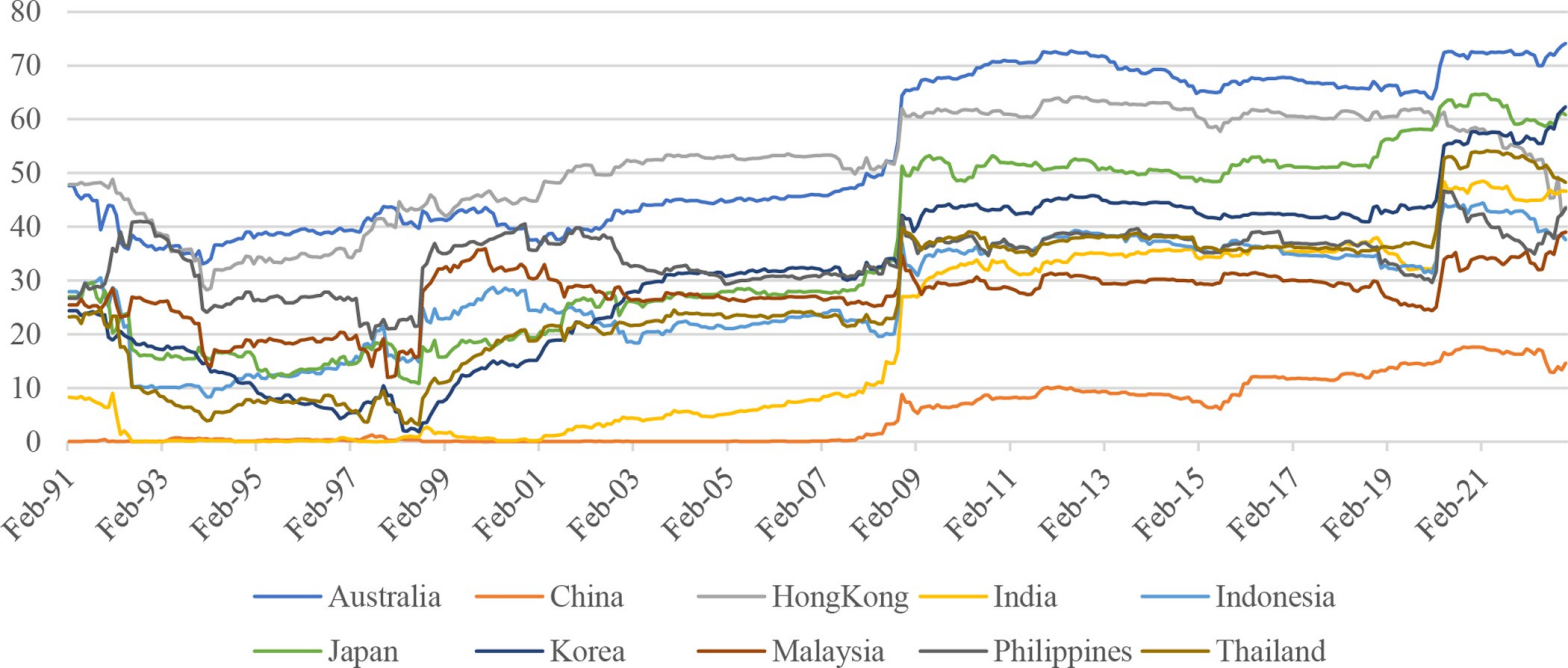
Spillovers from US’s S&P 500 to each Asia–Pacific stock market. Notes: Estimated based on TVP-VAR approach with the lag length of 1 (BIC) and one step-ahead forecast horizon.

Figs 2 and 3 above show the overall picture of the spillovers from the US stock market to the Asia-Pacific stock markets. Fig 4 shows the spillovers of the US stock market to individual markets in the Asia-Pacific region. In general, the influence of the US stock market on Asia-Pacific stock markets tends to increase gradually over time, especially after major crises such as the 1997 Asian financial crisis, the 2008 global financial crisis, the 2015 China stock market crash, and the COVID-19 pandemic in 2020. It is noted that there is a significant difference between the Asia-Pacific stock markets regarding the impact of the US stock market. Specifically, developed Asia-Pacific stock markets such as Australia, Hong Kong, and Japan are strongly affected by the US stock market. For example, around 70 per cent of the fluctuations in a unit of the Australian stock market return is from the return on the US stock market. This figure in Hong Kong and Japan are 60 per cent and 50 per cent, respectively. This difference can be attributed to the heterogeneity between these markets in several aspects, such as financial/trade openness [27] and the level of ICT development [31]. However, it is worth noting that after the COVID-19 pandemic, the spillover from the US market to the Hong Kong and Japanese markets has decreased and approached the level of emerging markets, while the transmission level from the US market to the Australian market remains relatively high compared to emerging groups. Furthermore, we also find that China was not affected by the US market before 2008 (spillover was almost at 0 per cent). However, the country is now affected by the US market after 2008. This finding is consistent with previous studies such as Dooley and Hutchison [58] and Mensi et al. [59], which document that increased coupling exists between the US and Chinese stock markets after the collapse of Lehman Brothers in 2008. However, the impact of the US stock market on China’s stock market is still relatively low compared to the impact of the US stock market on other countries in the same region.

**Fig 5:**
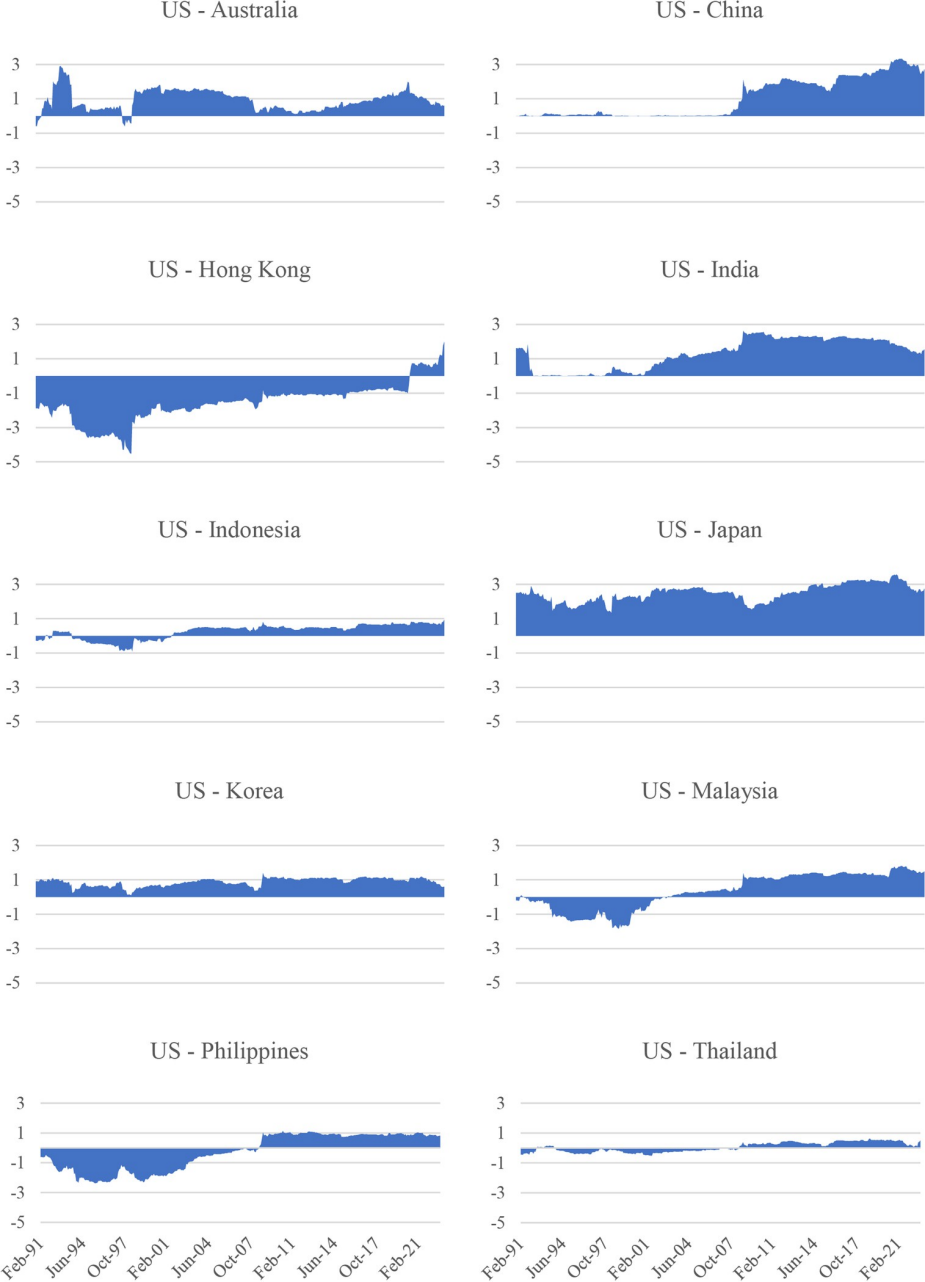
Net pairwise spillovers between the US stock market and each Asia–Pacific stock market. Notes: Estimated based on TVP-VAR approach with a lag length of 1 (BIC) and one step-ahead forecast horizon.

The net pairwise spillover between the US stock market and each Asia-Pacific stock market is the difference between the fluctuations transmitted from the US stock market return to each of the Asia-Pacific stock market returns and those transmitted from each Asia-Pacific stock market return to the US stock market return. A positive net pairwise spillover index implies that the US stock market is the net transmitter; otherwise, it is the net receiver. Fig 5 indicates that the spillover effect between the US stock market return and the stock market return in different Asia -Pacific stock markets is time-varying and different. The results show that the US stock market is mainly a net transmitter. However, the US stock market has generally been a net receiver of the market risk from the Hong Kong market until the emergence of the COVID-19 pandemic. In other words, the fluctuations of the Hong Kong stock market did not significantly affect the US stock market but were affected by the US stock market after the COVID-19 pandemic. China, India, Japan, and South Korea have been identified as net recipients from the US stock market during the research period, implying these markets are easily vulnerable to fluctuations in the US stock market. The significant and sudden change in the relationship between the Chinese and the US stock markets since the 2008 global financial crisis when the net pairwise spillover values increased from 0% in the previous period to 1.5% in 2009. The impact of the US market on the Chinese market has increased significantly since the protectionist trade policies in 2017 imposed by the US on China. The net pairwise spillover increases from 1.5% to 2.5% and has continued to increase until now.

### The effects of geopolitical risk and ICT on spillovers

We now examine the factors determining the spillovers from the US stock market to the Asia-Pacific stock markets. Table 4 presents the empirical results indicating that a 1% increase in geopolitical instability in Asia-Pacific countries leads to a reduction of 0.096% in spillovers from the US stock market to Asia-Pacific stock markets. This result validates our hypothesis 2a. Brennan and Cao [26] argue that there are differences in the available information between domestic and foreign investors. When geopolitical risk surges in the Asia-Pacific countries, it causes an asymmetry of information between local and foreign investors. This, in turn, reduces the cross-border investment activities of US investors and ultimately diminishes the spillovers of the US stock market onto the Asia-Pacific markets.

**Table 4:** The effects of geopolitical risk and economic policy uncertainty on the stock return spillovers from the US to Asia–Pacific stock markets.

	(1)	(2)	(3)	(4)
Local GPR	-0.097**			-0.097**
	(0.037)			(0.037)
US GPR		0.035***		0.011***
		(0.001)		(0.004)
US EPU			0.140***	0.157***
			(0.013)	(0.009)
ICT	0.038*	0.038*	0.038*	0.038*
	(0.021)	(0.021)	(0.021)	(0.021)
Financial Openness	2.602***	2.270***	2.270***	2.602***
	(0.484)	(0.453)	(0.453)	(0.484)
Trade Openness	0.043***	0.045***	0.045***	0.043***
	(0.010)	(0.010)	(0.010)	(0.010)
GDP growth	-0.047	-0.003	-0.003	-0.047
	(0.131)	(0.126)	(0.126)	(0.131)
Constant	22.230***	7.943***	4.569**	0.000
	(1.050)	(0.859)	(2.034)	(0.000)
Fixed effects	Country & Year	Country & Year	Country & Year	Country & Year
R-squared	0.796	0.792	0.792	0.796
Observations	297	297	297	297
Number of countries	10	10	10	10

Note: Driscoll-Kraay standard errors in paratheses. ***, **, and * indicate the significance level at 1 per cent, 5 per cent, and 10 per cent, respectively.

On the other hand, uncertainties originating from the United States, such as geopolitical risk and economic policy uncertainty, exhibit a positive association with the spillovers from the US stock market to the Asia-Pacific stock markets. This finding supports our hypothesis 2b, which suggests that a significant increase in uncertainties in the United States, such as changes in economic policy and geopolitical tensions, leads to a more extraordinary transmission of spillovers from the US stock market to the Asia-Pacific stock markets. This is because heightened uncertainties in the US have a detrimental effect on investment performance, causing investors to reallocate their portfolios away from the US. In addition, the results also reveal that both trade openness and financial openness are found to positively impact the spillovers, which supports hypothesis 3. Increased trade can cause a country to experience external shocks, significantly impacting the stock market and the economy [27]. Similarly, a higher degree of financial openness enhances the transmission of shocks between markets. If a country possesses a substantial amount of foreign assets, unexpected negative shocks from external sources can rapidly decline its balance sheet [28]. No evidence is found for the impact of real GDP growth on the spillovers. Finally, our empirical findings demonstrate that Information and Communication Technology (ICT) positively influences the spillover effects from the US stock market to the stock markets in the Asia-Pacific region, supporting our hypothesis 4. ICT enables faster and more efficient trading across various markets, reducing the time required to transfer information from one market to another. As a result, investors can promptly respond to fluctuations in the US market and adjust their investment strategies accordingly.

### Robustness checks

Our findings are based on adopting the TVP-VAR model with a 1-lag order determined by BIC criteria. Different lag order, i.e., lag 2, is now used as the robustness analysis. The results are presented in Appendix Fig A1 in S1 File. Additionally, different forecasted horizon (h = 10) is also used. The results are shown in Appendix Fig A2 in S1 File. We find that empirical findings largely remain unchanged compared to previous settings. Our empirical findings are robust with different settings regarding lag orders and forecasted horizons.

## Conclusions and policy implications

Our paper adopts the TVP-VAR connectedness approach to examine how the spillovers from the US stock market to the Asia-Pacific stock markets evolve from January 1991 to November 2022. This paper is the first to examine the determinants of the spillovers between stock markets. These determinants include geopolitical risk, economic policy uncertainty, trade openness, financial openness, GDP growth, and ICT. Furthermore, our study also assesses the effect of a series of major crises in history on the spillovers from the US stock market to the Asia-Pacific stock markets, including the 1997 Asian crisis, the 2008 global financial crisis, the 2015 China stock market crash, and the COVID-19 pandemic. Some findings are summarized as follows.

First, we find notable spillover effects of the US stock market on the stock markets in the Asia-Pacific region. This means that the impact of the US market on the Asia-Pacific stock markets is substantial, and the impact grows stronger as time passes, especially after major financial crises such as the 1997 Asian financial crisis, 2008 global financial crisis, 2015 China’s stock market crash, and most recently the COVID-19 pandemic. The 2008 crisis had the strongest impact, followed by the 1997 crisis, the COVID-19 pandemic, and finally 2015 China’s stock market crash. Second, we find that increases in Asia-Pacific countries’ geopolitical risk diminish the spillovers from the US to Asia-Pacific markets due to US investors’ concern about information asymmetry between domestic and foreign investors during heightened geopolitical risk in Asia-Pacific stock markets. By contrast, we find that heightened geopolitical risk and economic policy uncertainty in the US leads to an increase in spillovers from the US market to Asia-Pacific markets, consistent with the decoupling–recoupling hypothesis. Lastly, ICT, trade, and financial openness also contribute to the spillovers.

Managerial implications can be drawn from these findings. First, the Asia-Pacific markets are strongly affected by fluctuations in the US stock market. This impact is enlarged after major crises. Such a high spillover can limit the opportunity to diversify risk when investing in multiple markets, as downside risk in one market can quickly spread to another. As such, investors should be cautious during times of crisis, as opportunities to diversify risk are almost lost during these periods. Second, There are certain differences between markets regarding the extent to which they are affected. Accordingly, developed markets in the Asia-Pacific region are more vulnerable than emerging markets to shocks in the US stock market. As such, investors should look for investment opportunities in Asia-Pacific emerging markets if they diversify risks from the US market. As a final note, from a theoretical perspective, factors affecting the spillovers in this study, including geopolitical risk, ICT, trade openness, and financial openness, can be used to predict the trend of the US stock market’s influence on Asia-Pacific stock markets, which thereby can be used to develop appropriate investment strategies.

This study exhibits limitations. For further research directions in the future, while the current study examines the evolution of spillovers from the US stock market to the Asia-Pacific stock markets from January 1991 to November 2022, future research can extend the analysis to a longer period. This is important in the current context when geopolitical tensions between Russia-Ukraine and the US-China are still ongoing and constantly changing. This would provide insights into the long-term patterns and trends of spillover effects, helping to identify any structural changes or shifts in the relationships between these markets. The current study considers various determinants of spillovers, such as geopolitical risk, economic policy uncertainty, trade openness, financial openness, GDP growth, and ICT. However, future research could explore other factors that may impact spillover effects. By incorporating a broader set of determinants, a more comprehensive understanding of the complex mechanisms driving spillovers can be achieved.

## Supporting information

S1 File A list of the variables and their respective sources of data collection. (DOCX)

S1 Fig Spillover from the US stock market to all Asia–Pacific stock markets at different lag lengths. Notes: Estimated based on TVP-VAR approach.
(TIF)

S2 Fig Spillover from the US stock market to all Asia–Pacific stock markets at different forecasted horizons. Notes: Estimated based on TVP-VAR approach.
(TIF)
