# Supplementary material for: Market return spillover from the US to the Asia-Pacific Countries: The Role of Geopolitical Risk and the Information & Communication Technologies
Source: PLoS One. 2023 Dec 14;18(12):e0290680. doi: 10.1371/journal.pone.0290680 (PMC10721036; doi:10.1371/journal.pone.0290680)
Supplement: S1 File — (DOCX) [file pone.0290680.s001.docx]

**Appendix**

**Table A1.** A list of the variables and their respective sources of data collection

| **Variable** | **Description** | **Data source** |
| --- | --- | --- |
| Stock market indices | Australia All Ordinaries (Australia); Shanghai Composite (China); Hang Sheng (Hong Kong); S&P BSE Sensex (India); Jakarta Composite (Indonesia); Nikkei 225 (Japan); KOSPI (South Korea); FTSE Bursa (Malaysia); PSEi (Philippine); SET (Thailand); S&P 500 (US); Nasdaq Composite (US). | Refinitiv Eikon |
| Local GPR & US GPR | Country-specific geopolitical risk index (%) | Caldara and Iacoviello [12] via matteoiacoviello.com |
| US EPU | US Economic policy uncertainty index | Baker et al. [44] via policyuncertainty.com |
| ICT | Individuals using the internet (% of the population) | World Development Indicators |
| Trade openness | Trade openness index of each Asia-Pacific country as a per cent of gross domestic product (% of GDP) | World Development Indicators |
| Financial Openness | The financial openness index of each Asia-Pacific country | Chinn and Ito [60] via web.pdx.edu/~ito/Chinn-Ito_website.htm |
| Real GDP growth | Annual real GDP growth (%) | World Economic Outlook (IMF) |

**Figure A1.** Spillover from the US stock market to all Asia – Pacific stock markets at different lag lengths.

Notes: Estimated based on TVP-VAR approach.

**Figure A2.** Spillover from the US stock market to all Asia – Pacific stock markets at different forecasted horizons.

Notes: Estimated based on TVP-VAR approach.
