# Supplementary figures and images for: Market return spillover from the US to the Asia-Pacific Countries: The Role of Geopolitical Risk and the Information & Communication Technologies
Source: PLoS One. 2023 Dec 14;18(12):e0290680. doi: 10.1371/journal.pone.0290680 (PMC10721036; doi:10.1371/journal.pone.0290680)

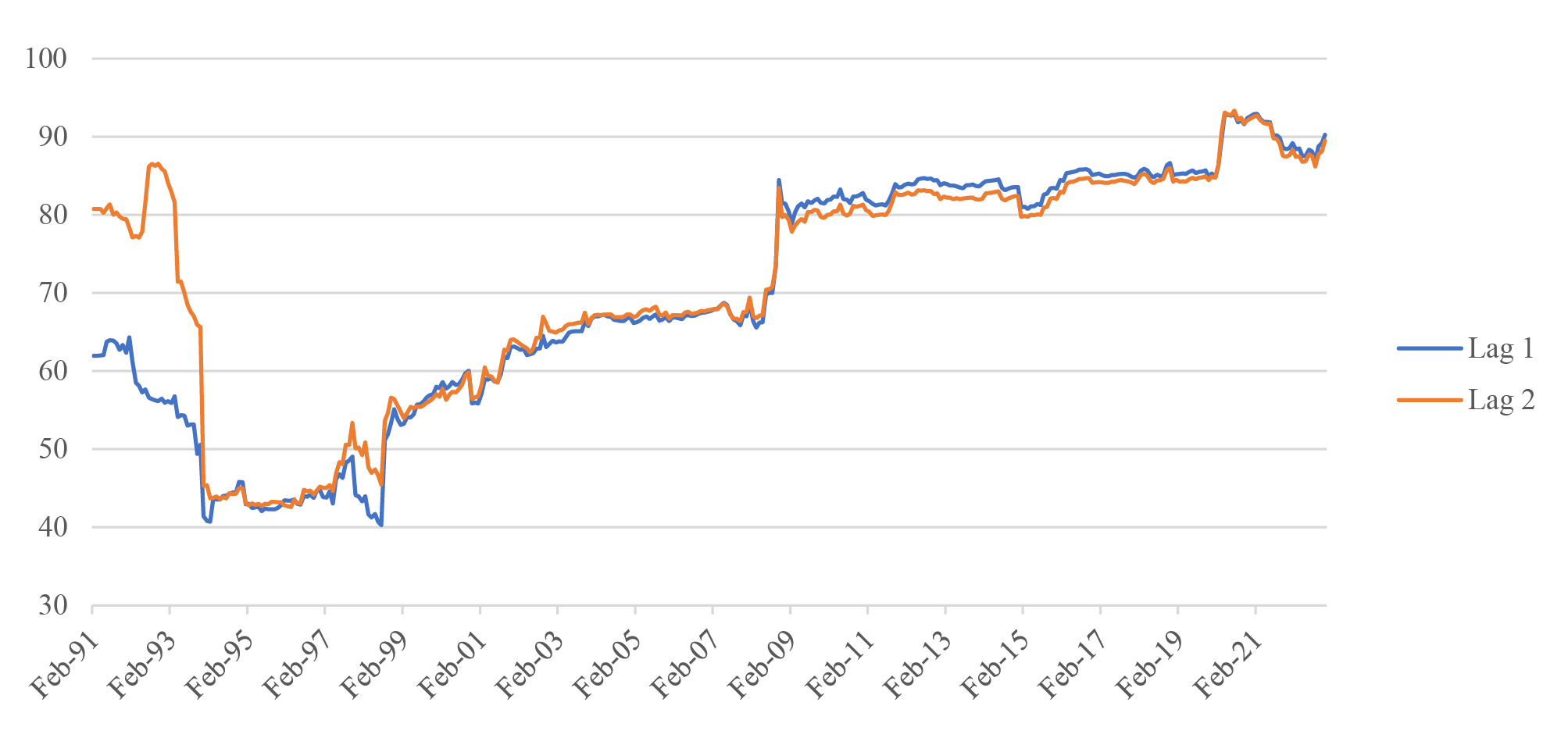

Supplement: S1 Fig — Notes: Estimated based on TVP-VAR approach. (TIF) [file pone.0290680.s002.tif]

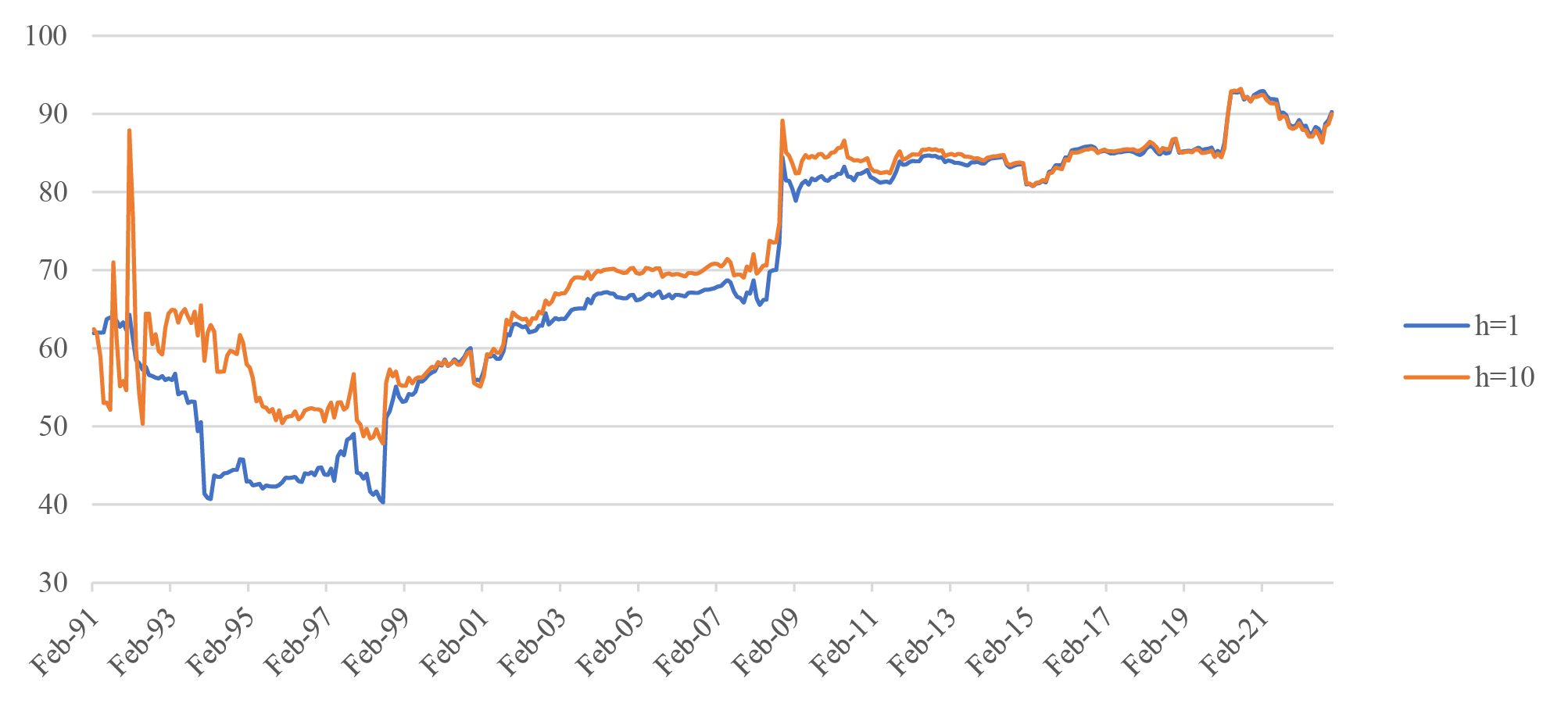

Supplement: S2 Fig — Notes: Estimated based on TVP-VAR approach. (TIF) [file pone.0290680.s003.tif]
